# Effect of dietary patterns and nutritional supplementation in the management of endometriosis: a review

**DOI:** 10.3389/fnut.2025.1539665

**Published:** 2025-03-12

**Authors:** Liyuan Zhou, Boya Liu, Xian Jian, Lili Jiang, Kuiran Liu

**Affiliations:** Department of Obstetrics and Gynecology, Shengjing Hospital of China Medical University, Shenyang, China

**Keywords:** endometriosis, inflammation, oxidative stress, dietary patterns, nutrition

## Abstract

Endometriosis is an estrogen-dependent chronic inflammatory disease which causes dysmenorrhea, chronic pelvic pain, and infertility in women of childbearing age, significantly impacting their quality of life and physical and mental health. The etiology of endometriosis remains unclear, with oxidative stress and inflammation currently thought to play pivotal roles in its pathophysiology. Epidemiological studies and clinical trials indicate that varying dietary patterns and specific nutrient supplementation can influence oxidative stress markers and levels of inflammatory factors and related pathways, potentially impacting the progression of endometriosis. In this review, we summarize the roles of oxidative stress and inflammation in endometriosis and thoroughly examine the current understanding of the effect of dietary patterns and nutrient supplementation in treating endometriosis. This study suggests that nutrients may prevent the occurrence of endometriosis by modulating levels of inflammatory factors, regulating angiogenesis, and influencing the metabolism of estrogen pathways. The findings might provide new insights into the treatment of endometriosis patients and the potential benefits of dietary patterns and nutrient supplementation in patients with endometriosis.

## 1 Introduction

Endometriosis is a common gynecological disorder characterized by the presence of endometrial tissue outside the uterus (1), typically presenting with severe pelvic pain and infertility, which affects 5–15% of reproductive-age women and as much as 3–5% of postmenopausal women (2). Endometriosis is a multifaceted, heterogeneous disease with no clear pathogenesis yet identified. Current research suggests that oxidative stress and inflammation play important roles in endometriosis. In the peritoneal fluid of patients with endometriosis, the concentration of numerous inflammatory factors, angiogenesis-related factors, and adhesion factors is elevated (3), and the activation of inflammation accelerates the progression of endometriosis (4). Endometriosis lesions stimulate the production of inflammatory factors and growth factors in the abdominal cavity. The persistent inflammatory response interacts with the central nervous system, resulting in chronic pain (5). Chronic pelvic pain significantly affects endometriosis patients’ quality of life and social functioning (6).

Dietary intervention and specific dietary patterns can influence the onset and outcomes of inflammation-related diseases, including cardiovascular disease, cancer, diabetes, and obesity (7). Different dietary patterns alter the levels of inflammatory factors and inflammation pathways in the body, affecting the occurrence of endometriosis. Ingestion of specific foods affects the levels of inflammatory factors, regulates angiogenesis, modulates the estrogen metabolism pathway in the body (8), and promotes or inhibits the development of endometriosis (9). The risk of endometriosis can be reduced by adjusting dietary intake, such as consuming nutrients with anti-inflammatory and antioxidant properties. Current research generally suggests that a high intake of green vegetables and fresh fruits (10), dairy products (11), legumes (12), polyphenols, and fish oil (13) significantly reduces the risk of endometriosis. Those with a high intake of red meat (14) and trans-fatty acids (15) have an increased risk of endometriosis. Women with endometriosis resort to non-medical methods to manage symptoms and enhance daily living (16). Self-care activities, complementary therapies, and positive doctor-patient relationships are critical components of endometriosis self-management.

**Figure 1:**
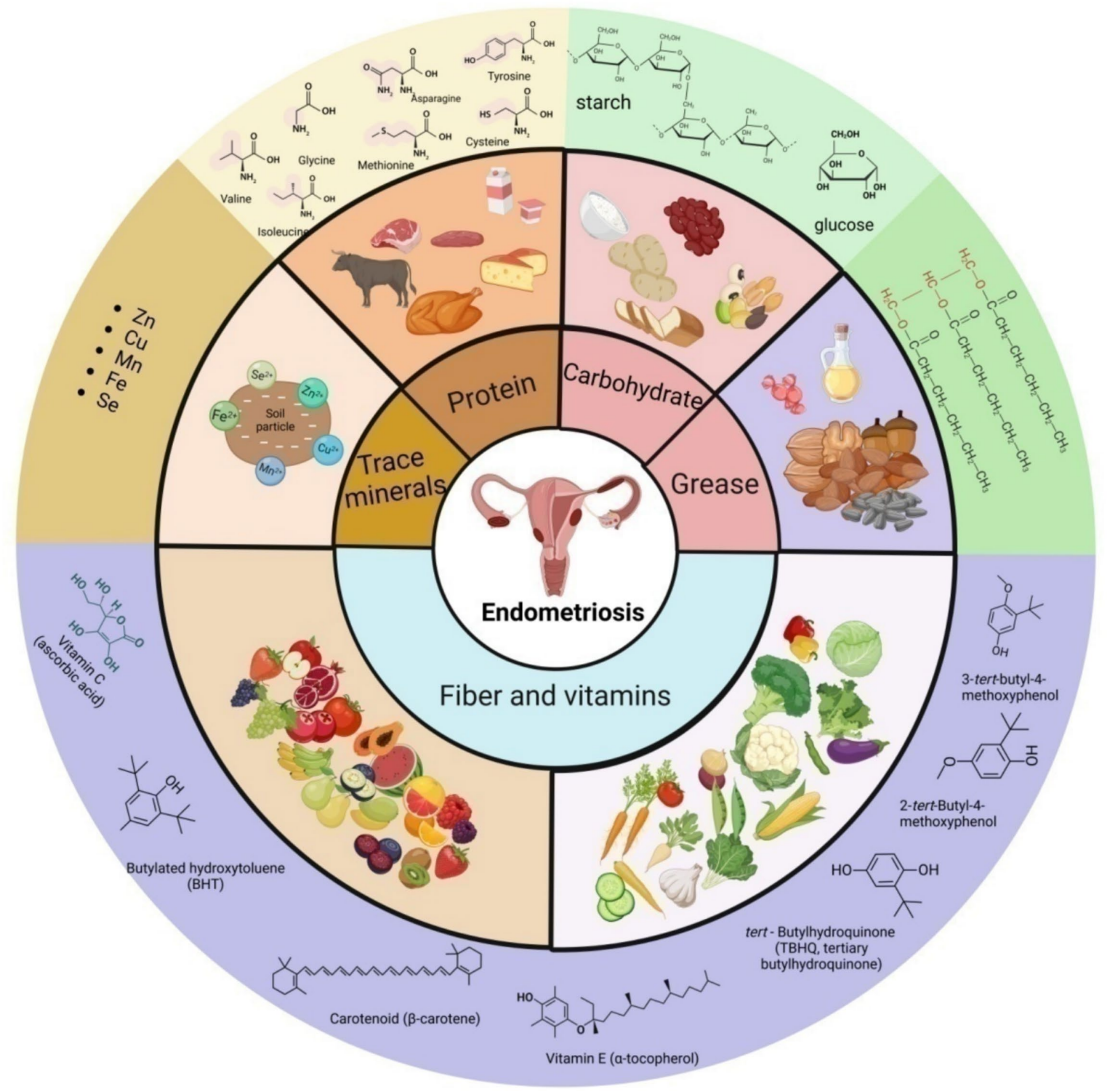
Schematic presentation of the main source, classification and some representative components of nutrition in endometriosis.

However, a few studies have yielded inconsistent results, and there is still a lack of large-scale, high-quality studies to verify the relationship among dietary patterns, nutrients, and endometriosis. The role of different dietary patterns and nutrients in endometriosis is still ambiguous. This review aims to provide an overview of oxidative stress and inflammation in endometriosis, and comprehensively summarize the effects and potential mechanisms of various dietary patterns and nutrients in endometriosis (Figure 1).

## 2 Oxidative stress and inflammation in endometriosis

There is evidence that endometriosis can be caused by retrograde menstruation (17), endometrial implantation, epithelial transformation (18), hormonal effects (19), and immune system dysfunction (20). Moreover, peritoneal fluid from people with endometriosis has unusual amounts of angiogenic and adhesion factors, as well as numerous inflammatory markers. This suggests that long-term inflammation plays a part in how endometriosis starts and gets worse (3). Endometriosis lesions stimulate the production of inflammatory cytokines and growth factors within the abdominal cavity, where the activation of inflammation further advances the progression of endometriosis (4).

**Figure 2:**
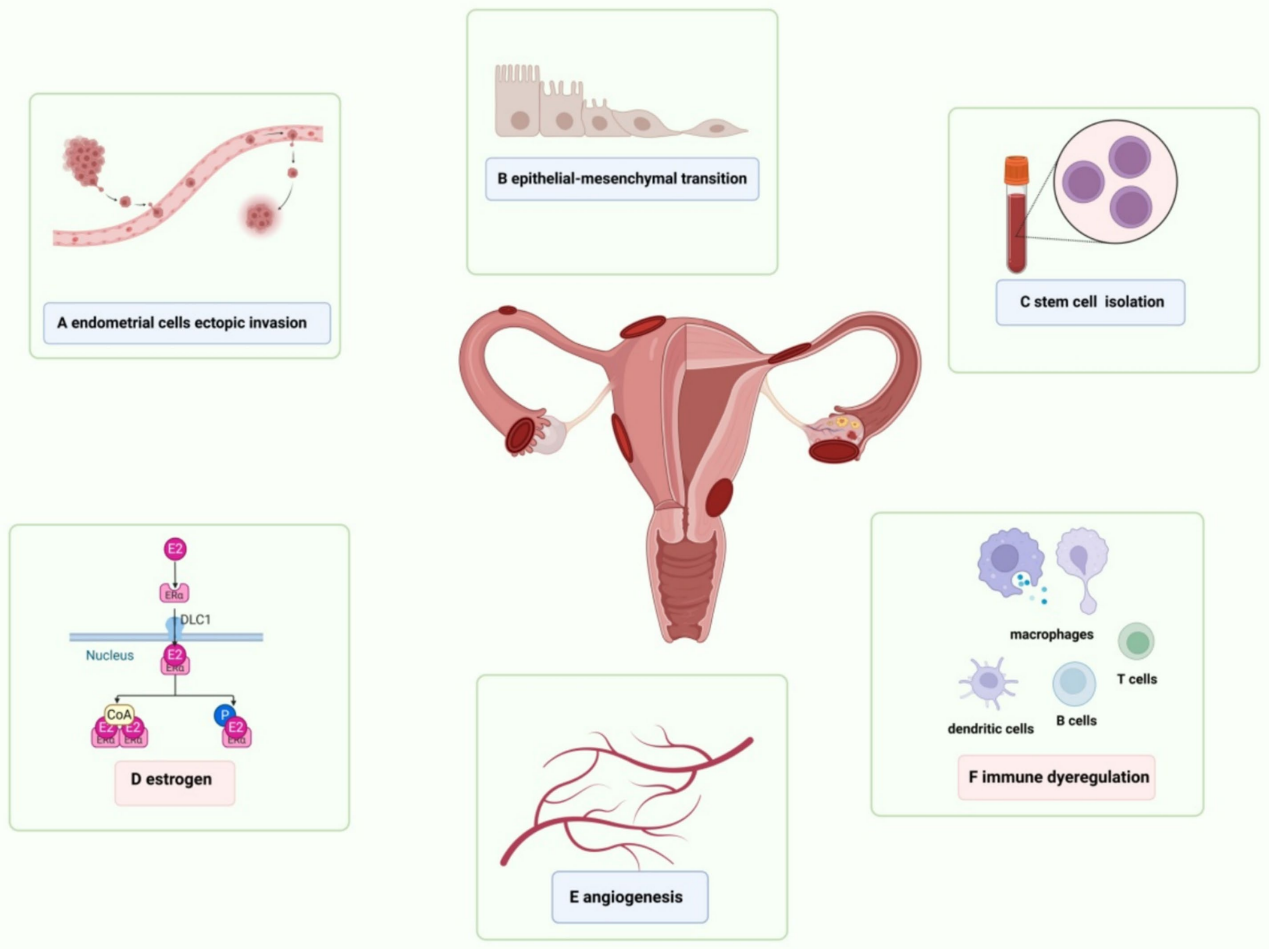
Schematic presentation of risk factors for endometriosis. **(A)** The exfoliated endometrial cells retrograde into the pelvic cavity and are implanted into the peritoneum and abdominal organs. **(B)** Epithelial-mesenchymal transformation is involved in the process of local inflammatory damage, repair, fibrosis, cell invasion and metastasis in endometriosis, as well as the formation of local lesions. **(C)** Adult stem cells are detected in endometrial suspension, which drive physiological endometrial regeneration and participate in the pathogenesis of endometriosis. **(D)** VEGF induces endothelial cell proliferation, migration and angiogenesis in endometriosis. **(E)** Elevated estrogen levels and the increased activity of estrogen receptors regulate immunity, inflammation and angiogenesis, and promote the continuous progression of endometrial lesions. **(F)** Abnormal activation of immune response and changes in peritoneal immune microenvironment are the basis of endometriosis.

Currently, it is believed that dietary ingredients with anti-proliferative, anti-inflammatory, antioxidant, analgesic, and estrogen-reducing properties can reduce the incidence of endometriosis. Nevertheless, the exact modus operandi remains obscure. Consequently, we have meticulously updated and comprehensively rearticulated these two mechanisms, with a particular focus on innovative and all-encompassing methodologies (Figure 2).

### 2.1 The role of oxidative stress in endometriosis

Macrophages, red blood cells, and apoptotic endometrial tissue transplanted into the peritoneal cavity via retrograde menstruation serve as inducers of oxidative stress (21). The release of hemoglobin and iron from red blood cells activates macrophages, leading to the production of inflammatory factors including interleukin-1, interleukin-6, and tumor necrosis factor-alpha (22) and fostering the formation of damaging reactive oxygen species (23). The iron storage levels (ferritin load) in the peritoneal macrophages of patients with endometriosis are significantly elevated compared to the control group (24). The continuous increase in iron content triggers oxidative stress, resulting in local damage to the peritoneal mesothelium and creating adhesion sites for ectopic endometrial cells (25). Iron overload further exacerbates the condition by stimulating cell proliferation, thereby advancing endometriosis (26, 27).

ROS are intermediates generated during normal oxygen metabolism in the human body, with a delicate balance maintained between pro-oxidants and antioxidants in healthy individuals. Mitochondria generate ROS via the electron transport chain, a process termed oxidative phosphorylation (OXPHOS) (28). An excess of oxidants triggers oxidative stress (29), leading to a significant elevation in mitochondrial superoxide levels within ectopic endometrial tissue (30), which alters mitochondrial structure, particularly by expanding the surface area of the cristae. This influences OXPHOS function and leads to increased ROS production (31). Excess ROS regulates cell proliferation (32) and leads to the overactivation of nuclear factor kappa-B (NF-κB) by IL-1 and TNF-α. NF-κB enhances the invasive and adhesive capacities of endometriotic cells to the peritoneal surface by regulating the expression of matrix metalloproteinases, thereby stimulating angiogenesis and inflammation (33). Although mitochondria are the primary ROS producers, they are vulnerable to ROS attacks (30).

### 2.2 The role of inflammation in endometriosis

The coordinated action of macrophages, endometrial cells, endothelial cells, and activated lymphocytes, along with cytokines and chemokines, alters the abdominal immune microenvironment, facilitating pathological processes including cell proliferation, angiogenesis, lesion adhesion, growth, and invasion (34). Macrophage numbers significantly rise in peritoneal fluid and ectopic endometrium (35), and co-culturing macrophages with endometrial stromal cells (ESCs) enhances the proliferative and invasive capacities of ESCs (36). Co-culturing ESCs with monocyte-derived macrophages and NK cells from endometriosis patients showed that the interaction between ESCs and macrophages downregulates NK cell cytotoxicity by enhancing the release of IL-10 and TNF-β (37), potentially allowing endometrial fragments to evade immune clearance (38). Studies have reported that patients with endometriosis have an increased presence or activation of B lymphocytes (39). Anti-endometrial antibodies have been detected in the serum and peritoneal fluid of patients with endometriosis (40). These autoantibodies can stimulate the immune system, causing persistent inflammation, advancing endometriosis, and contributing to the formation of the inflammatory microenvironment.

The endometriosis microenvironment may enhance ERK activity in endometriotic cells. TNFα and IL-1β are capable of activating ERK and inducing the expression of IL-8 and IL-6 (41). The chemokine MCP1 also greatly increases the production of PGE2 (42), VEGF, IL-8, and MCP-1 in human endometriotic cells through a pathway specific to ERK (43).

The PI3K/AKT/mTOR pathway regulates cell growth, proliferation, differentiation, and apoptosis (44). Membrane-bound phosphoinositide 3-kinase (PI3K) is the most common mediator of mTOR activation, forming the core of the PI3K/AKT/mTOR pathway along with AKT (45). When PI3K is turned on, it causes PDK1 to be phosphorylated by AKT. This then turns on downstream mTOR receptors by interacting with TSC2 (46). The TSC complex, consisting of TSC1 and TSC2, interacts with GTPase-Ras, acting as a negative regulator of mTOR activity (47). When TSC2 is phosphorylated by kinases such as AKT, it dissociates from the complex, leading to the inactivation of the complex and the subsequent activation of mTORC1 by Rheb, which is bound to GTP (48). When activated, mTORC1 activates numerous downstream proteins, promoting protein synthesis and cell growth. Additionally, mTORC1 activates HIF-alpha through VEGF, serving as the primary angiogenic switch (49), thereby inducing new angiogenesis. LncRNA IGF2-AS facilitates the progression of endometriosis by targeting the miR-370-3p/IGF2 axis and activating the PI3K/AKT/mTOR signaling pathway (50). Interventions targeting HIF-1α and mTOR pathways may emerge as potential therapeutic targets for endometriosis (51).

Additionally, E2 regulates protein and DNA synthesis in uterine epithelial cells via the PKC/ERK/mTOR pathway, ultimately controlling cell proliferation (52). In endometrial stromal cells (ESC), TNFα-induced activation of the estrogen receptor ER leads to heightened ERK activation (53). Lipoprotein A4 suppresses inflammation and enhances autophagy via the AhR/mTOR/AKT pathway, thereby inhibiting endometriosis (54).

IL-37b, a unique member of the IL-1 family, may inhibit lesion growth by regulating proliferation, invasion, angiogenesis, and inflammation through the AKT and ERK1/2 signaling pathways (55). Recombinant human IL-37 enhances the Th1/Th2 ratio by inducing dendritic cell maturation, thus curbing the progression of endometriosis in mouse models (56). Exogenous IL-1β increases the production of nerve growth factor in primary endometrial stromal cells in endometriosis. This is associated with the onset of endometriosis and the concomitant pain (57). Interleukin-33 is a member of the IL-1β family. In mouse models that did not have IL-33, the size of endometriotic lesions was greatly reduced (58). When injected into a monkey model of endometriosis with its long-acting recovery antibody (AMY109), IL-8, a potent chemoattractant for angiogenic factors and immune cells, can inhibit neutrophil recruitment to endometrial lesions and suppress their production of monocyte chemotactic protein-1, thereby ameliorating endometriosis inflammation and fibrosis (59). IL-17 is upregulated in the serum, peritoneal fluid (PF), and endometrial lesions of patients with endometriosis (60), regulating the recruitment and M2 polarization of peritoneal macrophages in endometriosis (61).

**Figure 3:**
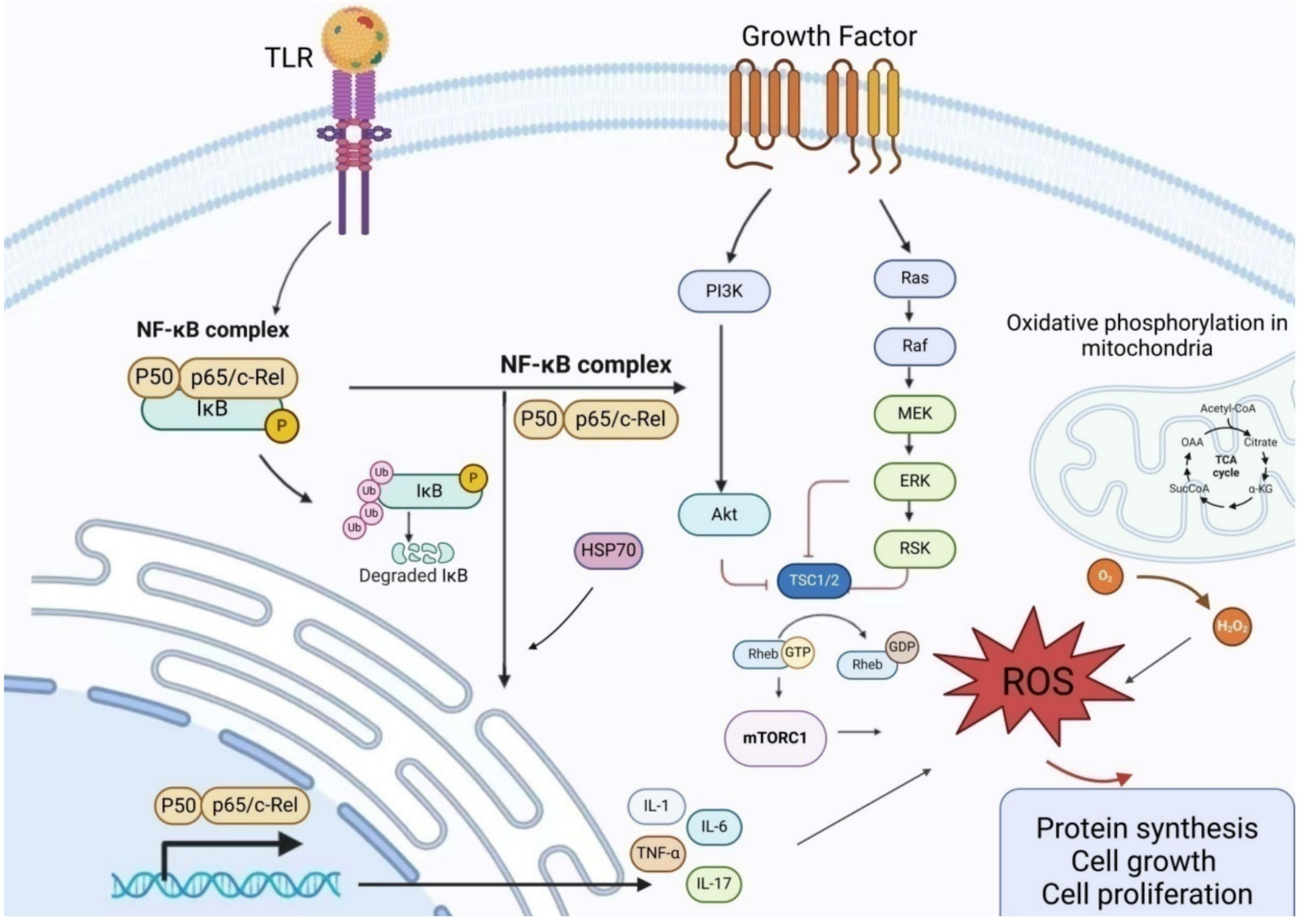
Schematic presentation of the mechanism of action of inflammatory factors in endometriosis NF-κB promotes the production and release of inflammatory factors by regulating the expression of matrix metalloproteinases. Excessive ROS regulates cell proliferation and induces excessive activation of NF-κB by IL-1 and TNF-α, thereby attacking mitochondria. The PI3K/AKT/mTOR pathway regulates cell growth, proliferation, differentiation, and apoptosis. Upon PI3K activation, AKT phosphorylation of PDK1 is stimulated, and the interaction between AKT and TSC2 activates downstream mTOR receptors. The TSC complex, consisting of TSC1 and TSC2, interacts with GTPase-Ras to serve as a negative regulator of mTOR activity. When TSC2 is phosphorylated by kinases (such as AKT), it dissociates from the complex, rendering it inactive. This leads to Rheb binding to GTP, activating mTORC1, and triggering an oxidative stress response. AKT, Protein Kinase B; ERK, Extracellular Signal-Regulated Kinase; GDP, Guanosine DiPhosphate; GTP, Guanosine TriPhosphate; MEK, Mitogen-Activated Protein Kinase; mTORC1, Mechanistic Target of Rapamycin Complex 1; NF-κB, Nuclear factor kappa-B; PI3K, Phosphatidylinositol 3-Kinase; RAF, RAF kinase; RAS, Rat Sarcoma Virus Oncogene; ROS, Reactive oxygen species; TRL, Toll-like receptor; TSC, Tuberous Sclerosis Complex.

HSP-70, a chaperone protein within the heat shock protein family, is produced by macrophages, smooth muscle cells, endometrial cells, dendritic cells, and vascular endothelial cells. It significantly stimulates the production of vascular endothelial growth factor, interleukin-6, and TNF-α in women with endometriosis. Furthermore, HSP-70 facilitates Toll-like receptor 4 (TLR4)-mediated growth of endometrial cells (62). The decrease in HSP-70 may facilitate unrestricted nuclear translocation of NF-κB, resulting in targeted transcription (Figure 3).

## 3 Effect of dietary patterns in endometriosis

Endometriosis is characterized by hormone dependency and a high recurrence rate, necessitating lifelong management. Dietary intervention is currently one of the main methods for self-management for endometriosis patients, with numerous studies indicating that a higher intake of vitamin-rich foods reduces the risk of endometriosis (63–65). Excessive intake of red meat is associated with increased levels of estrogen sulfate, leading to higher levels of steroids and inflammatory factors that influence the progression of endometriosis (66, 67). Consuming dairy products, fruits, legumes, red meat, and potatoes is significantly associated with a reduced risk of endometriosis. Consuming fried potatoes, on the other hand, has a positive correlation with the risk of endometriosis. Intake of animal proteins, EPA, MUFA, and oleic acid is associated with a reduced risk of endometriosis (68). Those in the top fifth with the highest intake of trans-unsaturated fats have a 48% higher likelihood of being diagnosed with endometriosis (15). In the absence of ovarian dysfunction and insulin resistance in mice, high dietary fat intake increases the risk of endometriosis (69).

Most of the aforementioned studies focus on the association between specific foods and endometriosis, failing to establish a link between the participants’ overall dietary nutrient intake and the condition. When other factors are taken into account, the findings may be contradictory. Schwarz (70) found that the beneficial effects associated with fruit fiber vanished after adjusting for the healthy diet index. Furthermore, an elevated risk of endometriosis was noted when combining the total intake of vegetables and cruciferous fiber. Currently, there are no specific dietary recommendations for endometriosis patients. We have outlined the key features of prevalent dietary patterns and explored the connections between various dietary patterns and endometriosis.

### 3.1 Mediterranean diet

Mediterranean diet (MedDiet) includes eating a lot of fresh, seasonal, minimally processed plant-based foods, like vegetables, fruits, legumes, potatoes, bread, nuts, seeds, and other grains; a lot of olive oil (OO), especially virgin olive oil (VOO) and extra-virgin olive oil (EVOO), which is the main source of fat; some dairy products, like cheese and yogurt; and some poultry and fish, which are main sources of long-chain polyunsaturated fatty acids (PUFAs) (71), especially omega-3 fatty acids. Typically, the caloric intake from fat should not exceed 30%, with less than 8–10% from saturated fats.

The various nutrients in MedDiet, such as vitamins, minerals, polyphenols, fibers, nitrates, PUFAs, and monounsaturated fatty acids (MUFAs), are biologically active compounds that are beneficial to health (72, 73). They have synergistic and interactive effects in reducing inflammation (73) and affect different inflammatory markers in the body, such as IL-6 or TNF-α (74, 75). Flavonoids in grains, vegetables, fruits, and olive oil have potentially beneficial effects, including free radical scavenging, anti-inflammatory effects, and anti-Aβ neurotoxicity. Dietary polyphenols can also effectively combat ROS, reduce oxidative damage to genetic material, and enhance the antioxidant capacity of endothelial cells (76). The polyphenols in olive oil regulate the inflammation by inhibiting NF-κB (77). Resveratrol can improve the antioxidant status of Parkinson’s disease rats and reduce dopamine loss (78). When several polyphenols are used in combination, they exhibit stronger antioxidant capacity in terms of their activity (79). Hydroxytyrosol, which is found in extra virgin olive oil, can lower inflammation by stopping cyclooxygenase-2 (COX-2) and inducible nitric oxide synthase from working (80). HT can also reduce superoxide ions and inhibit the excessive secretion of the important inflammatory mediator prostaglandin E2 in the human body (81).

Olive oil that is high in MUFA is crucial for keeping the lipid profile of mitochondrial membranes in check so that they can fight oxidative damage and age-related problems (82). People with a higher intake of olive oil have more MUFA in their mitochondrial membranes, while those who mainly consume sunflower oil have more omega-6 PUFA. These changes are consistent with the level of oxidative damage; namely, compared to those who consume sunflower oil, those who consume OO have fewer hydroperoxides in their body tissues. Therefore, a diet rich in olive oil produces membranes with less PUFA, which weakens the increase in lipid peroxidation (83). HT and oleuroside can reduce oxidative stress and optimize mitochondrial function (84). Fish oil is a beneficial source of omega-3 PUFAs. Giving older mice fish oil for 21 days increased the amount of omega-3 PUFA derivatives in their brains, which led to better mitochondrial function and ATP production (85). Fish oil omega-3 supplementation may reduce endometrial implant growth and inflammatory factor production, particularly in patients with stage III or IV endometriosis (13). Supplementing omega-3 polyunsaturated fatty acids alleviates pain symptoms related to endometriosis (86).

It is now believed that the long-term Mediterranean diet has a potential protective effect on cardiovascular diseases, stroke, obesity, diabetes, hypertension, malignant tumors, allergic diseases, as well as Alzheimer’s disease and Parkinson’s disease (87). Patients with endometriosis who follow the Mediterranean diet can reduce inflammatory factors in their bodies and alleviate pain symptoms (88).

### 3.2 Ketogenic diet

The characteristic of a low-carbon ketogenic diet is the consumption of extremely high-fat and low-carbohydrate diets, with carbohydrate intake ≤10% of energy consumption (89). This dietary pattern forces the system to shift from glucose metabolism to fatty acid metabolism, thereby producing ketones. A ketogenic diet increases low-density lipoprotein cholesterol in healthy, young, normal-weight women (90). Low carbohydrate intake causes physiological ketosis in patients with polycystic ovary syndrome, which reduces circulating insulin levels, lowers IGF-1 levels, and inhibits stimulation of androgen production, including in the ovaries and adrenal glands. Reducing circulating lipids, low-grade inflammation, and oxidative stress also helps prevent cardiovascular complications (91, 92). Low-fat and low-carbohydrate diets significantly reduce the concentrations of several serum inflammatory markers, such as TNF-α, IL-6, IL-8, MCP-1, etc. (93). After 21 days on a carefully formulated ketogenic diet (WFKD), women show improvements in body composition, blood pressure, and blood sugar; increased ketone bodies; and improvements in some, but not all, cholesterol markers (94). WFKD can improve fasting blood glucose, insulin resistance, weight, and body composition in women with stage IV metastatic breast cancer. WFKD serves as an adjunctive therapy, playing a positive role (95).

A low-carbohydrate ketogenic diet can alleviate chronic musculoskeletal pain, improve blood biomarkers, and enhance the quality of life of patients (96). The ketogenic diet regulates gut microbiota and short-chain fatty acids, which are associated with Alzheimer’s disease markers in subjects with mild cognitive impairment (97).

### 3.3 Low FODMAP diet

FODMAP stands for fermentable oligosaccharides, disaccharides, monosaccharides, and polyols. These osmotic carbohydrates are non-absorbable and undergo bacterial fermentation in the small intestine, leading to functional gastrointestinal symptoms such as cramps, bloating, and diarrhea (98). A short-term moderate-low-FODMAP diet can significantly alleviate gastrointestinal symptoms, with significant reductions in pain, bloating, diarrhea, and fullness scores (99).

The symptoms of endometriosis are closely related to other chronic diseases such as irritable bowel syndrome (IBS) (100), inflammatory bowel disease (IBD), and celiac disease (CD) (101). A low-FODMAP diet reduces irritable bowel symptoms in patients with endometriosis (102, 103). After 6 months on a low-FODMAP diet, women with endometriosis reported reduced pain and improved quality of life (104). After 3 months on a low-FODMAP diet, significant improvements were observed in all chronic pelvic pain, dysmenorrhea, and both intestinal and extraintestinal symptoms (105).

### 3.4 Gluten-free diet

GFD involves avoiding foods containing gluten (such as wheat, barley, spelled kham, rye, and triticale). The diet typically includes carbohydrates without gluten (GF) sources (such as rice, quinoa, corn, buckwheat, and legumes) and/or industrial GF products. Gluten-free products are typically high in saturated fats, sugars, and salts (106) and low in protein, fiber, and vitamins (107). Pain symptoms of endometriosis were alleviated after 12 months on a gluten-free diet (108). Celiac disease (CD) is an autoimmune disorder affecting 1% of the population, causing reversible inflammation of the small intestine mucosa and accompanied by acute symptoms such as diarrhea, constipation, bloating, nausea, and vomiting. A lifelong gluten-free diet (GFD) is a treatment for Crohn’s disease (109). However, some studies suggest that the increased gut side effects of a gluten-free diet may lead to adverse health outcomes (Table 1) (101).

**Table 1:** The effect of different dietary patterns on endometriosis.

Dietary patterns	Effect	Clinical research of endometriosis
Mediterranean diet (87)	1. Reducing inflammation (73) 2. Oxidative stress (76) 3. Optimize mitochondrial function (84)	1. Inflammatory factors ↓ (88) 2. Pain symptoms ↓ (88)
Ketogenic Diet	1. Circulating lipids, low-grade inflammation, and oxidative stress ↓ (92) 2. TNF-α, IL-6, IL-8, MCP-1 ↓ (93)	1. Increase LDL cholesterol ↑ (90) 2. Decreased triglycerides, total cholesterol, and low-density lipoprotein ↓ (91); High-density lipoprotein ↑ 3. Estradiol, progesterone, and SHBG were elevated ↑ (91)
Low FODMAP diet	1. Pain, abdominal distension of IBS and IBD ↓ (101, 163)	1. Abdominal distension and diarrhea ↓ (102, 103) 2. Pelvic pain ↓; Abdominal distension ↓ (104)
Gluten-free diet	1. Irritable bowel symptoms ↓ (164) 2. Diarrhea, constipation, bloating ↓ (109)	1. Dysmenorrhea ↓ (108)

IL-6, Interleukin-6; IL-8, Interleukin-8; LDL, Low density lipoprotein; MCP-1, Monocyte chemotactic protein-1; TNF-α, Tumor necrosis factor-α.

## 4 Effects of nutrient supplementation in endometriosis

The pathogenesis of endometriosis remains under investigation, prompting many affected women to adopt dietary interventions to manage symptoms and enhance daily living (16). A cross-sectional study revealed a dietary shift among endometriosis patients, with increased consumption of vegetables, fruits, grains, legumes, and fish, coupled with a decrease in dairy products, soy-based foods, and high saturated fat intake (110). An Italian study reported that 76% of women with endometriosis employed self-management strategies, with 44% opting for dietary changes, achieving a high score in dietary management effectiveness (111).

Various dietary patterns influence the onset of endometriosis by modifying the levels of inflammatory factors and the inflammation pathways within the body. Consuming specific foods influences the levels of inflammatory factors, regulates angiogenesis, affects the estrogen metabolism pathway (8), and can promote or inhibit the progression of endometriosis. Supplementation with antioxidant vitamins can effectively reduce dysmenorrhea severity, ameliorate chronic pelvic pain, and enhance the quality of life for these patients (112). The risk of endometriosis can be mitigated by dietary adjustments, particularly the consumption of nutrients with anti-inflammatory and antioxidant properties (113). We summarized the antioxidant capacity of various nutrients and their roles in animal models of endometriosis and endometriosis patients.

### 4.1 Vitamin C and vitamin E

VC can inhibit ROS and AKT/mTOR signaling, thereby preventing mesenchymal stem cell aging (114). Additionally, VC inhibits HIF1a transcription and increases HIF1 alpha-hydroxylase activity, leading to mitochondrial activation (115) and antioxidant effects. In mouse models, VC contributes to anti-ovarian aging by influencing collagen synthesis, angiogenesis, aging, cell proliferation, and differentiation (116). Intravenous vitamin C treatment prevents the induction of endometrial implants while inhibiting the regression of existing ones in mouse models (117). VE significantly lowers serum levels of CRP and IL-6 (118). Vitamin E enhances antioxidant status by increasing TAC levels in postmenopausal women, offering greater anxiety relief compared to the placebo group (119). Vitamin C and E supplementation alters the expression and production of vascular endothelial growth factor genes in patients with endometriosis (120), effectively reducing the severity and improving pelvic pain (121).

### 4.2 Vitamin D

VD can inhibit the activation of nuclear factor kappa B, reduce the expression of IL-1β, TNF-α, and IL-8, and decrease prostaglandin activity (122). In a mouse model, supplementation with VD induces an anti-inflammatory phenotype in macrophages and exhibits anti-proliferative properties (123). Serum levels of 25-hydroxyvitamin D3 negatively correlate with endometriosis (124). People with endometriosis who take vitamin D have much less pelvic pain and lower levels of hs-CRP, TAC, and total cholesterol/high-density lipoprotein cholesterol ratio (125). Supplementation with VD reduces pelvic pain in young patients with endometriosis (126) and ameliorates immune-inflammatory biomarkers in young postmenopausal women (127).

### 4.3 Epigallocatechin-3-gallate

The antioxidant and anti-inflammatory properties of tea polyphenols may have potential therapeutic effects on endometriosis (128). The amount of mRNA for nuclear factor kappa B and mitogen-activated protein kinase 1 goes up when epigallocatechin-3-gallate (EGCG) is present (129). Furthermore, in mice, EGCG selectively inhibits angiogenesis and blood perfusion in endometriotic lesions, leading to their regression (130). Pro-EGCG greatly slows down the development, growth, and formation of new blood vessels in experimental endometriosis, showing that it can both protect cells from damage and stop the growth of new blood vessels (131). Some genes, like vascular endothelial growth factor C (VEGFC) and the tyrosine kinase receptor VEGF receptor 2 (VEGFR2), are turned down by EGCG (132). EGCG downregulates VEGFC/VEGFR2 signaling through pathways involving c-JUN, interferon-γ, matrix metalloproteinase-9, and chemokine ligand 3, thereby inhibiting endothelial proliferation, inflammation, and cell migration. EGCG prevents the activation of MAPK and Smad signaling pathways stimulated by TGF-β1 in both endometrial and endometriotic stromal cells by a large amount. Animal experiments indicate that EGCG can prevent the progression of fibrosis in endometriosis (133).

### 4.4 Melatonin

In endometriosis, there is an increased expression of melatonin receptors (134). Melatonin mitigates oxidative stress, inflammation processes, and cell apoptosis by upregulating the Nrf2 signaling pathway and downregulating COX-2 protein levels (135), as well as SOD, GPx, CAT, and Bcl-2 activities (136). Melatonin inhibits the development of endometriosis by disrupting mitochondrial function and regulating siRNA expression in mouse models (137). Melatonin treatment reduces MMP-3 activity in mouse endometriosis models and facilitates endometriosis regression through caspase-3-mediated pathways, enhancing endometriotic cell apoptosis (138). Melatonin lowers the activity and expression of proMMP-9, which has anti-inflammatory effects and lessens the damage caused by peritoneal endometriosis in mice (139). A clinical study suggests that oral administration of 10 mg of melatonin before bedtime during menstruation provides better analgesic relief for dysmenorrheal (140).

### 4.5 Quercetin

Quercetin can reduce the expression of IL-6, IL-1β, and TNFα (141). Quercetin induces p21 CDK inhibitors that decrease pRb phosphorylation by capturing E2F1, thus inhibiting G1/S cell cycle progression. Low doses of quercetin induce mild DNA damage and activate Chk2, a key regulator of quercetin-induced p21 expression. Quercetin also lowers the levels of cyclin B1 and CDK1, which prevent transcription. This suggests that quercetin can stop the cell cycle from moving forward in physiologically relevant doses (142). Quercetin treatment effectively suppresses the growth of endometrial lesions in mice with endometriosis (143).

Quercetin induces the downregulation of ERK1/2, P38 MAPK, and AKT signaling molecules, leading to DNA fragmentation, mitochondrial membrane potential loss, and ROS production, thereby inducing cell apoptosis (144). Following intraperitoneal quercetin injection in mice, Ccnd1 mRNA expression was significantly reduced compared to the control group, leading to G0/G1 cell cycle arrest and reduced cell proliferation, accompanied by increased apoptosis of VK2/E6E7 and End1/E6E7 cells. In mouse models, the activity of oxidative stress markers is reduced *in vivo* through the Nrf2 signaling pathway (145). For example, it can prevent LPS from causing oxidative stress in the jejunum by activating the MAPK/Nrf2 signaling pathway. This can fix the damage that LPS does to the mitochondria in the jejunum and increase the expression of mitochondrial DNA copy number-related genes like COX1, ATP6, and ND1 (146).

### 4.6 Resveratrol

Resveratrol (RSV), a polyphenol found in red wine, regulates NF-κB activity (147). It inhibits Th17 cells, decreasing the production of the inflammatory factor IL-17 (148). When resveratrol is added to the stromal cells of women with endometriosis, the levels of IGF-1 and HGF go down (149).

Resveratrol decreases the concentrations of MCP1, VEGF (150), IL-6, IL-8, and TNF α in peritoneal fluid *in vitro* and in animal models (151), inhibiting angiogenesis and inflammation to regress endometriotic lesions (152). It reduces the invasiveness of endometrial stromal cells and curbs the progression of endometriosis in nude mouse models (153). Metastasis-associated protein 1 (MTA1) increases epithelial-mesenchymal transition (EMT) through its connection with ZEB2. Resveratrol suppresses ectopic lesions’ growth and MTA1 and ZEB2 expression. MTA1 may be a target for resveratrol (154). Taking 400 mg of resveratrol every day for 12–14 weeks lowers the amounts of matrix metalloproteinases MMP-2 and MMP-9 in endometriosis patients’ serum and peritoneal fluid, which lowers inflammation (155).

### 4.7 Curcumin

E2 levels in endometriosis lesions are significantly elevated; supplementing with curcumin can notably decrease estradiol levels, thereby inhibiting the growth rate of endometriosis (156). Curcumin can additionally suppress endometrial cells in endometriosis by downregulating the vascular endothelial growth factor (157). Curcumin contributes to the treatment of mouse models for endometriosis by modulating the HIF signaling pathway, ameliorating local hypoxia, and diminishing inflammation (158). Curcumin therapy reduces endometriosis in mice by preventing NFκB translocation and suppressing MMP-3 expression. It primarily enhances cell apoptosis in endometriosis via the cytochrome c-mediated mitochondrial pathway (159). Curcumin slows the progression of endometriosis in mice by inhibiting MMP-2 activity (160). Curcumin blocks endometriosis by reducing matrix metalloproteinase-9 activity (161). Curcumin supplementation can ameliorate oxidative stress levels (MDA and TAC) in postmenopausal women and lower inflammatory biomarkers (hs-CRP) (Table 2) (119).

**Table 2:** The effect of nutrients in endometriosis.

Nutrient	Molecular mechanism	Experimental animal models	Clinical research
VC	1. ROS and AKT/mTOR signal transduction ↓ (114) 2. Hif1-α hydroxylase activity ↑; HIF1-α transcription ↓; Mitochondrial activation ↑ (115) 3. Change VEGF gene expression and production (120)	1. Rat: Anti-ovarian aging (116) 2. Rat: Inhibit the growth of endometriosis lesions (117)	Vitamin C and vitamin E supplements: 1. Dysmenorrhea ↓ (121) 2. Systemic oxidative stress index ↓ (121)
VE	CRP and IL-6 ↓ (118)		TAC ↑, antioxidant ↑, and anxiety ↓ in postmenopausal women (119)
VD	1. Activation of NF -κB ↓; IL-1β ↓, IL-8 ↓; prostaglandin activity ↓ (122) 2. IL-6 ↓, TNF-α1 ↓ (165)	Rat: Anti-proliferative properties and induces an anti-inflammatory phenotype in macrophages (123)	1. Dysmenorrhea ↓ (126, 166) 2. Immuno-flammatory biomarkers ↓ (127) 3. Negatively associated with endometriosis in American women (124) 4. hs-CRP ↓, TAC ↑, and dysmenorrheal ↓ (125)
*Viburnum opulus* L.	Antioxidant capacity ↑ (167)	Rat: Induced lesion reduction (168)	NA
Melatonin	1. Nrf2 signaling pathway ↑ COX-2 protein ↓ (135) 2. SOD, GPx, CAT and Bcl-2 activity ↑ (136) 3. The melatonin receptor ↑ (134)	1. Rat: Disrupt mitochondrial function and regulate tiRNA to inhibit (137) 2. Rat: MMP-3 ↓, apoptosis ↑, Bcl-2 ↓, Bax ↑, caspase-9 activity ↑ (138) 3. Rat: proMMP-9 ↓, TIMP-1↑ (139)	1. Dysmenorrhea ↓ (140)
EGCG (128)	1. Inhibits estrogen-induced activation of endometrial cells (130) 2. NFκB ↑, mitogen-activated protein kinase1- mRNA ↑ (129)	1. Rat: angiogenesis ↓, lesion ↓ (131) 2. Rat: VEGF-C/VEGFR2 expression ↓, lesion ↓, reduced VEGFR2 and ERK activation (132) 3. Rat: antifibrosis (133) 4. Rat: Selective inhibition VEGF-A (129)	NA
Trans-fatty acid	Tumor necrosis factor system hyperactive: TNFR, IL-6and CRP ↑ (169)	Rat: TNFα and redox status ↑, oxidative stress ↑, estrogen receptor 1 isoform and progesterone receptor ↓ (69)	The highest fifth of intakes were 48 percent more likely to be diagnosed with endometriosis (15)
Dairy		Rat: TNF-α ↓, IL-6 ↓ (170)	Dose-dependent reduction of endometriosis risk (11, 171)
Quercetin	1. IL-6, IL-1β, and TNF-α ↓ (141) 2. Induce p21 CDK inhibitor, inhibit the G1/S cell cycle; induced mild DNA damage and Chk2 activation; inhibits G2/M cell cycle, Transcriptional inhibition by inhibit NF-Y (142) 3. Inhibit VK2/E6E7, End1/ E6E7 and induce cell cycle arrest; ERK1/2 ↓, P38 MAPK ↓, and AKT ↓ (144)	1. Rat: NF-κB ↓, oxidative stress ↓ (145) 2. Broiler chickens: oxidative stress ↓ by MAPK/Nrf2; Mitochondrial damage ↓; Nrf2 ↓; MAPK ↓, JNK ↓, ERK ↓, and p38MAPK ↓ (146) 3. mRNA of Ccnd1 ↓; VK2/E6E7↑ and End1 / E6E7 apoptosis ↑; miR-503-5p and miR-546↑ (144)	NA
Resveratrol (RSV)	1. Th17 cells ↓, IL-17 ↓ (148) 2. IGF-1 ↓, HGF ↓ (149)	1. Invasiveness ↓ (153) 2. Rat: Inflammatory cytokine ↓, COX enzyme ↓, prostaglandin ↓, inflammation ↓ (152) 3. Rat: MTA1 ↓, ZEB2 ↓ (154)	mRNA and protein levels of MMP-2 and MMP-9 ↓ (155), inflammation ↓ (155).
Curcumin	1. Estradiol ↓ (156) 2. VEGF ↓ (157)	1. Rat: HIF-1α mRNA and protein synthesis ↓; IL-1β, IL-6, and VEGFA ↓ (158) 2. NFκB ↓, MMP-3 ↓ (159) 3. MMP-2 ↓ (160) 4. MMP-9 ↓ (161)	MDA ↓, hs-CRP ↓ and TAC ↑ (119) in menopausal women

Bcl-2, B-cell lymphoma-2; CAT, Catalase; COX2, Cyclo-oxygenase2; CRP, C-reactive protein; ERK, Extracellular regulated protein kinases; GPx, Glutathione Peroxidase; HGF, Hepatocyte growth factor; HIF1-α, Hypoxia inducible factor-1α; hs-CRP, High sensitivity C-reactive protein; IGF-1, Insulin-like growth factor-1; MAPK, Mitogen-activated protein kinases; MTA1, Metastasis associated protein 1; MMP, Matrix metalloproteinase; NF-κB, Nuclear factor kappa-B; Nrf2, Nuclear factor erythroid 2-related factor 2; p21 CDK inhibitor, P 21 cyclin dependent kinase inhibitor; ROS, Reactive oxygen species; SOD, Superoxide Dismutase; TAC, Total antioxidant capacity; TIMP, Tissue inhibitor of metalloproteinase; Th17, T helper 17 cell; VEGF, Vascular endothelial growth factor; VEGFR2, Vascular Endothelial Growth Factor Receptor 2; ZEB2, Zinc Finger E-Box Binding Homeobox 2.

## 5 Conclusion

Oxidative stress and inflammatory factors play crucial roles in endometriosis. This article summarizes the roles of oxidative stress and inflammatory factors in endometriosis and reviews the risk factors of endometriosis. Additionally, we have summarized the current mainstream dietary patterns and the impact of common nutrients on endometriosis. Supplementing nutrients can inhibit the progression of endometriosis in various ways, such as reducing levels of inflammatory factors, decreasing oxidative stress responses, inhibiting cell proliferation, and reducing angiogenesis. The Mediterranean dietary paradigm, renowned as a quintessential anti-inflammatory dietary regimen, is capable of curtailing the levels of inflammatory mediators within the body and mitigating the symptoms experienced by patients afflicted with endometriosis. Nutrients endowed with anti-inflammatory properties, including vitamins (such as vitamin C and vitamin E), quercetin, resveratrol, curcumin, and epigallocatechin-3-gallate (EGCG), hold the potential to diminish the lesions associated with endometriosis and thus exhibit latent therapeutic implications. Analyzing endometriosis patients’ dietary traits and formulating new preventive dietary strategies (162) promise to mitigate disease incidence and lessen the patients’ burden. The anti-inflammatory and antioxidant properties of nutrients may be key areas of focus. Currently, there are no dietary guidelines for endometriosis patients, and previous studies were limited in sample size and primarily descriptive. Existing research on dietary patterns and the role of nutrients in endometriosis is insufficiently comprehensive. There is a pressing need to delve deeper into the pathogenesis of endometriosis and the impact of varied dietary patterns, aiming to identify optimal dietary regimes for these patients. This approach holds significant promise for alleviating symptoms and halting disease progression.

## Glossary

COX2: Cyclo-oxygenase2
ESCs: Endometrial stromal cells
ERK: Extracellular regulated protein kinases
HIF-α: Hypoxia inducible factor-1 α
HT: Hydroxytyrosol
MAPK: Mitogen-activated protein kinases
MCP-1: Monocyte chemotactic protein-1
MUFA: Monounsaturated fatty acids
mtDNA: Mitochondria DNA
NGF: Nerve growth factor
NF-κB: Nuclear factor kappa-B
OO: Olive oil
OS: Oxidative stress
OXPHOS: Oxidative Phosphorylation
PKC: Phosphokinase C
PI3K: Phosphate inositol 3 kinase
PUFA: Polyunsaturated fatty acids
RSV: Resveratrol
ROS: Reactive oxygen species
TSC: Tuberculous protein sclerosis complex
TLR4: Toll like receptor 4
VEGF: Vascular endothelial growth factor
WFKD: Well-formulated ketogenic diet
